# Commentary on: effect of vitamin D on insulin resistance and anthropometric parameters in type 2 diabetes; a randomized double-blind clinical trial

**DOI:** 10.1186/2008-2231-21-19

**Published:** 2013-03-08

**Authors:** Azar Baradaran

**Affiliations:** 1 Department of Clinical Pathology, Isfahan University of Medical Sciences, Isfahan, Iran

## 

Dear Editor-in-Chief

I read with interest the published article in the esteemed journal by Heshmat et al., entitled “effect of vitamin D on insulin resistance and anthropometric parameters in type 2 diabetes; a randomized double-blind clinical trial” [1]. The study has focused to investigate the effect of injection of vitamin D on insulin resistance and anthropometric parameters in type 2 diabetes mellitus (T2DM) [1]. Heshmat et al. studied 42 diabetic patients with similar baseline characteristics in two groups; intervention group with single intramuscular injection of 300,000 IU of vitamin D_3_ and the placebo group. They found that, 3 months after vitamin D injection, HbA_1_c, anthropometric factors and homeostasis model assessment (HOMA) index in intervention group stayed constant, however, serum 25- OHD_3_ was significantly increased. They suggested that, single injection of vitamin D was not accompanied by better diabetes control and improvement of insulin resistance [1]. Similar to this study, we conducted a double blind randomized clinical trial on 60 T2DM patients who were divided into 2 groups with 30 patients in each [2]. Group 1 were treated with oral Vitamin D, and group 2 were treated with placebo drug. After 3 months of treatment intervention, no significant difference of serum HbA_1_c and lipids between two groups was found. We concluded that, weekly vitamin D supplementation for 12 weeks had not significant decremented effect on HbA_1_c and lipid profiles [2].

Studies concerning the beneficial effects of vitamin D supplementation on improvement of diabetes or improvement of insulin sensitivity are limited and revealed different results. To find, whether receiving vitamin D_3_ (4000 IU/d) is associated with improved markers of insulin sensitivity and resistance, and also reduced inflammation in obese adolescents, Belenchia et al. studied participants who have supplemented with vitamin D_3_ for 6 months. They found a significant increase in serum 25-hydroxyvitamin D concentrations after this period. However, there were no significant differences in body mass index, serum inflammatory markers or plasma glucose concentrations in comparison to control group. Moreover, inflammatory markers remained unchanged [3]. Meanwhile, in the study conducted by Lim et al., on 1080 non-diabetic Korean subjects, it was found that 25(OH)D baseline is associated with the incidence of T2DM in high-risk subjects for up to 5 years of follow-up, independently of obesity, baseline insulin resistance, and β cell function [4]. Furthermore, to test the association of low plasma 25-hydroxyvitamin D with increased risk of T2DM in the general population, Afzal et al. measured 25-hydroxyvitamin D level in 9841 participants from the general population, of whom 810 developed type 2 diabetes during 29 years of follow-up. They found the association of low plasma 25-hydroxyvitamin D with increased risk of T2DM [5].

Prevalence of type 2 diabetes mellitus (T2DM) is increasing worldwide [6-11] and based on increasing evidence from animal and human studies, vitamin D deficiency is now regarded as a potential T2DM risk factor [12-18]. Hence, the present data is not convincing and further studies with large sample sizes are needed to show the definite effect of vitamin D supplementation on control of diabetes and its risk.
